# Using Theory to Explore the Determinants of Medication Adherence; Moving Away from a One-Size-Fits-All Approach

**DOI:** 10.3390/pharmacy5030050

**Published:** 2017-08-30

**Authors:** Claire Easthall, Nina Barnett

**Affiliations:** 1 School of Healthcare, University of Leeds, Woodhouse Lane, Leeds LS2 9JT, UK; 2 London North West Healthcare NHS Trust & NHS Specialist Pharmacy Service, Pharmacy Department, Northwick Park Hospital, Watford Road, Harrow HA1 3UJ, UK; nina.barnett@nhs.net

**Keywords:** medication adherence, health behaviors, health coaching, motivational interviewing, psychology

## Abstract

Non-adherence to prescribed medicines has been described as “a worldwide problem of striking magnitude”, diminishing treatment effects and wasting resources. Evidence syntheses report current adherence interventions achieve modest improvements at best, and highlight the poor progress toward the longstanding aim of a gold-standard intervention, tailored to meet individual need. Techniques such as motivational interviewing and health coaching, which aim to facilitate patient-centred care and improve patient resourcefulness, have shown promise in supporting adherence, especially in patients with psychological barriers to medicine-taking, such as illness perceptions and health beliefs. Despite a plethora of research, there is little recognition that the nature and complexity of non-adherence is such that a one-size-fits-all approach to interventions is never likely to suffice. This commentary re-visits the call for adherence interventions to be tailored to meet individual need, by considering what this means for day-to-day practice and how this can be achieved. It provides an update on advances in psychological theory to identify the root cause of an individual’s non-adherence to encourage matching of provided adherence support. It also provides a practical perspective by considering exemplars of innovative practice and evaluating the day-to-day practicalities of taking a novel approach.

## 1. Introduction

The World Health Organization (WHO) has described medicine non-adherence as “a worldwide problem of striking magnitude”; 30–50% of patients prescribed medicines for long-term conditions do not take their medicines as prescribed [1]. Medicine non-adherence has been associated with sub-optimal treatment outcomes [2], increased morbidity and mortality [3,4], wasted healthcare resources [2], and as a predictor of 30-day hospital readmission [5]. Despite both the extent and significance of medicine non-adherence, a gold-standard adherence intervention remains elusive. The most recent Cochrane review of medicine adherence highlights a paucity of effective adherence interventions and lack of progress in the field; interventions which improve adherence do so by marginal gains at best and improvements in both adherence and clinical outcomes are rare [6]. The authors highlight that a lack of grounding in theory and failure to target interventions toward the specific determinants of adherence may be causative in this lack of progress. This commentary provides an overview of advances in the application of psychological theory to medication adherence and explores exciting opportunities to address the deficits highlighted by the Cochrane review. It also provides a practical perspective by considering how these advances in thinking can be applied to time-limited, routine consultations seen in day-to-day practice.

## 2. Medication Adherence: Current Understanding and Interventions

Medication adherence is a complex health behavior and vast literature is available to describe its predictors and influencing factors [1,2,7,8]. Socio-demographic factors, such as age and gender, tend to be weak predictors of adherence, whereas factors such as illness perceptions and health beliefs have long been recognized as more powerful predictors [7,9]. The seminal work of Horne and colleagues describes the balance between a patient’s perceived necessity for their medicines and concerns about this [9,10]. Whilst influential in this field of research, the necessity-concerns framework only concerns patients’ conscious decisions to adhere and is, thus, less useful in exploring practical barriers to adherence. Non-adherence can broadly be viewed as an intentional or unintentional behavior. Intentional non-adherence concerns perceptual barriers; the patient has the ability to adhere but makes a conscious decision not to. Determinants of this behavior include illness perceptions and health beliefs, which in turn, reduce a patient’s **motivation** to adhere. Unintentional non-adherence concerns more practical barriers to adherence; physical and cognitive deficiencies, such as dexterity or memory problems, which impede medicine-taking. Patients who are unintentionally non-adherent are therefore willing to take their medicines as prescribed, but are unable to do so, as practical impediments reduce their **capability** or **opportunity** to adhere. When considering medicine adherence, we of course think of oral dosage forms, such as tablets and capsules, but non-adherence to prescribed medicines includes all dosage forms. Non-oral dosage forms such as creams and inhalers may be subject to more practical barriers to adherence as errors in administration can occur, but it is pertinent to remember that irrespective of dosage form and route of administration, a myriad of intentional and non-intentional adherence behaviors will exist. 

In the quest for a gold-standard adherence intervention, many researchers have looked to specific techniques to improve adherence. Compliance aids exemplify this; long considered a panacea for adherence support, these devices stand to benefit patients with a limited range of adherence barriers, primarily those with deficits in cognition or dexterity. Importantly, patients whose non-adherence is intentional in nature will not benefit from these costly devices [11]. More recently, there has been a marked interest in the use of reminder apps to support medication non-adherence, again these interventions stand only to support patients in whom memory is a determinant of adherence. A patient unwilling to take their medicines due to health beliefs and illness perceptions will gain no support from this one-size-fits-all approach, which disregards the complexity of non-adherence and the plethora of influencing factors. 

Routine care is predominated by educational and behavioral interventions to support adherence, which offer practical solutions, such as reminder prompts, adherence aids, regimen simplification, and increasing patient knowledge about their medicines. Whilst potentially beneficial for instances of unintentional non-adherence, these strategies fall short of an effective method to support patients who have made a conscious decision not to adhere. Most pharmacists should feel confident in overcoming practical barriers to adherence such as cognitive or dexterity issues, yet addressing the more perceptual barriers to adherence such as health beliefs, self-confidence, and motivation, presents more of a challenge. ‘Cognitive-based behavior change techniques’, such as motivational interviewing, health coaching, and other approaches that aim to change patients’ motivation, thoughts, and feelings toward adherence have shown promise as tools in our armory of approaches to support adherence. Evidence from a comprehensive meta-analysis suggests that these interventions may improve adherence beyond the educational and behavioral interventions currently delivered in standard care and most importantly, can be delivered with efficacy by routine healthcare providers such as pharmacists [12]. The key of course is to recognize that motivational interviewing would be completely inappropriate for a patient whose non-adherence was determined by simple practical issues, such as dexterity problems. Yet again, we return to our core message that there is no one-size-fits-all approach to adherence and that the intervention selected must be matched to meet individual need. However, these cognitive-based approaches offer an exciting tactic, especially for the more challenging instances of intentional non-adherence. A pertinent question therefore concerns how these newer techniques can be incorporated into our-day-to-day practice as pharmacists; Section 4 of this commentary provides an invaluable perspective on this. 

Provision of education has potential utility in resolving instances of both intentional and unintentional non-adherence, but should be applied with caution in patients unwilling to take their medicines as prescribed. The pioneers of motivational interviewing, Rollnick and Miller, provide invaluable guidance on this matter, commenting that provision of persuasive advice can evoke further resistance to change, particularly in patients who are not ready to change their behavior [13]. The point made here is a game changer, highlighting that well-intended advice-giving can actually push patients further away; when seen by the patient as ‘nagging’, the provision of generic education may just frustrate the patient even more and heighten their resistance to change. The pertinent message here is that the provision of education and advice-giving should not be deployed until the nature of an individual’s non-adherence has been identified. It should then be provided in a tailored-fashion to address the individual need; once again a one-size-fits-all approach is inappropriate and can even do more harm than good. 

Consider, for example, two patients, both non-adherent with their inhaled corticosteroid for asthma. Without exploring the reasons for non-adherence in depth, it is impossible to select an appropriate intervention to encourage improved adherence. Patient A may be non-adherent due to dexterity problems that prohibit use of the inhaler device, whereas patient B may have misconceptions about the medicine, believing, for example, that they will develop a body-builder-like physique from using a steroid-based inhaler. In both instances, SMS or app-based reminder prompts will not help and may, in fact, do more harm than good, leaving both patients unsatisfied, disenfranchised, and with a sense of feeling misunderstood. Similarly, provision of generic education focused purely on the benefit of using the inhaler as persuasive advice to encourage adherence will not help. Patient A, who wants to use their inhaler but cannot, most likely knows why they should be using their inhaler and thus, receipt of generic advice is likely to, once again, induce feelings of alienation, frustration and possibly even shame. Patient B, on the other hand, also knows why they should be using their inhaler, but has chosen not to due to fear of side effects, generic advice giving will therefore precipitate similar negative feelings and may evoke further resistance to change. However, had the nature of non-adherence been identified before intervening, advice-giving in both instances could have been targeted to meet individual need. Given the complexity of adherence determinants, due consideration, therefore, needs to be given to strategies to identify barriers to adherence at an individual level. 

## 3. Medication Adherence from a Theoretical Perspective: Identifying Barriers to Change

Whilst useful in considering the determinants of adherence from a broad perspective, the distinction between intentional and unintentional non-adherence is imperfect. Behaviors such as forgetting to take medicines may appear to be unintentional; however, when explored in greater depth, patients may ‘forget’ to take their medicines when this is not a priority for them. Subsequently, a seemingly unintentional behavior (forgetting) may actually stem from determinants grounded in intentional behaviors, even if the patient is not consciously aware of this. Understanding the root cause of non-adherence, beyond the arbitrary distinction of intentional and unintentional behaviors is, therefore, pertinent in the quest to move forward. 

Whilst non-adherence has long been considered in terms of intentional and unintentional behaviors, exploration of adherence determinants in terms of capability, opportunity, and motivation represents a new way of thinking. Michie and colleagues have revolutionized the application of psychological theory to behavior change, offering an accessible and intuitive approach to explore the determinants of behavior and match evidence-based behavior change techniques to these. The COM-B model [14] (Capability, Opportunity, Motivation–Behavior) states that an individual must have adequate capability, opportunity, and motivation for a behavior (such as medicine-taking) to take place. If there are deficits in any one of these areas, the behavior is unlikely to occur. In relation to medicine-taking, an individual’s capability to adhere may be influenced by physical factors such as dexterity or eyesight, and psychological factors such as knowledge and memory. A patient with memory impairment would, thus, lack the psychological capability to adhere, whereas a patient with dysphagia may lack the physical capability to adhere. Opportunity to adhere can be considered in terms of physical and social opportunities. Patients lacking a physical opportunity to adhere may include those with difficulties accessing medicines due to environmental constraints, such as difficulties getting to pharmacies, or lack of healthcare insurance. Social opportunities to adhere include issues such as availability of social support, which has been demonstrated to be a strong predictor of adherence [15]. The fascinating concept of social norms is also covered by this aspect; fear of stigma associated with medicine-taking is problematic for conditions such as HIV [16], but also in medicine-taking adolescents who fear being different to their peers [17]. Motivation to adhere can be split into reflective motivation, where factors such as self-confidence and beliefs are critical to success, and automatic motivation, which concerns the more visceral responses to medicine-taking, such as emotions, which are often overlooked. ‘Emotions’ have recently been highlighted as important determinants of adherence [18].

The COM-B model allows the determinants of behaviors such as medicines-taking to be explored in more depth than the binary model of intentional and unintentional non-adherence and thus facilitates the identification of the root cause of non-adherence. The Theoretical Domains Framework (TDF) [19,20] is closely linked to the COM-B model, but explores behavioral determinants at an even greater depth. Both the TDF [19,20] and COM-B model [14] have been applied to medication adherence in recent literature [18,21,22,23,24,25,26], highlighting notable progress in this field of research. Whilst other psychological models of behavior have relevancy to medication adherence, the application of these models tends to be from a theoretical perspective and thus lacks utility in practical application in supporting intervention design [27,28]. The unique strength of the TDF and COM-B models is that both have been linked to evidence-based behavior change techniques [14,29]. This means the seeds are sown for matching adherence interventions to the specific determinants of behavior.

## 4. Using Health Coaching to Support Adherence through Person-Centred Care: Examples from Practice

Health coaching, an umbrella term for a number of behavior change methods, uses techniques from psychology and performance coaching to help the patient identify a health-related goal and develop their own options for solving the issues that are raised. Whilst applied to medicine adherence in a number of clinical settings, including diabetes [30], health coaching is new to pharmacy practice. Though not specifically focused on medication adherence, a 2015 report commissioned by NHS Health Education East of England makes a strong case for the effectiveness of healthcare provider-led health coaching to support behavior change across a diverse range of long-term conditions [31]. A 2014 systematic review of health coaching for adults with chronic conditions [32] reported one study with significant positive outcomes for medication adherence [30]. More recent studies [33,34] highlight the application of health coaching to support patients with diabetes and suggest that an updated review of health coaching for medicines adherence in long-term conditions may be warranted. Other behavior change techniques such as motivational interviewing [35], adherence therapy [36], and cognitive behavioral therapy [37] are also being used in pharmacy practice, though as yet none are commonly utilized. 

Health coaching has been used in UK hospital pharmacy practice to develop a coaching approach to pharmacy consultations [38]. An accredited health coaching program was first delivered by one of the authors (NB) in collaboration with The Performance Coach (now TPC Health) to pharmacy staff in 2014 where training in health coaching techniques was provided during a formal two-day program. This training was followed by local support sessions to embed the skills in practice and facilitate adoption into routine care. This program of work is currently being evaluated but preliminary data suggests that a combination of support is desirable, including formal training, group workshops and one-to-one practice based visits [39,40]. The coaching approach was central to the development of an integrated medicines management service [41] and health coaching training has now been commissioned for healthcare professionals working in the Northwest London area [39].

**Figure 1:**
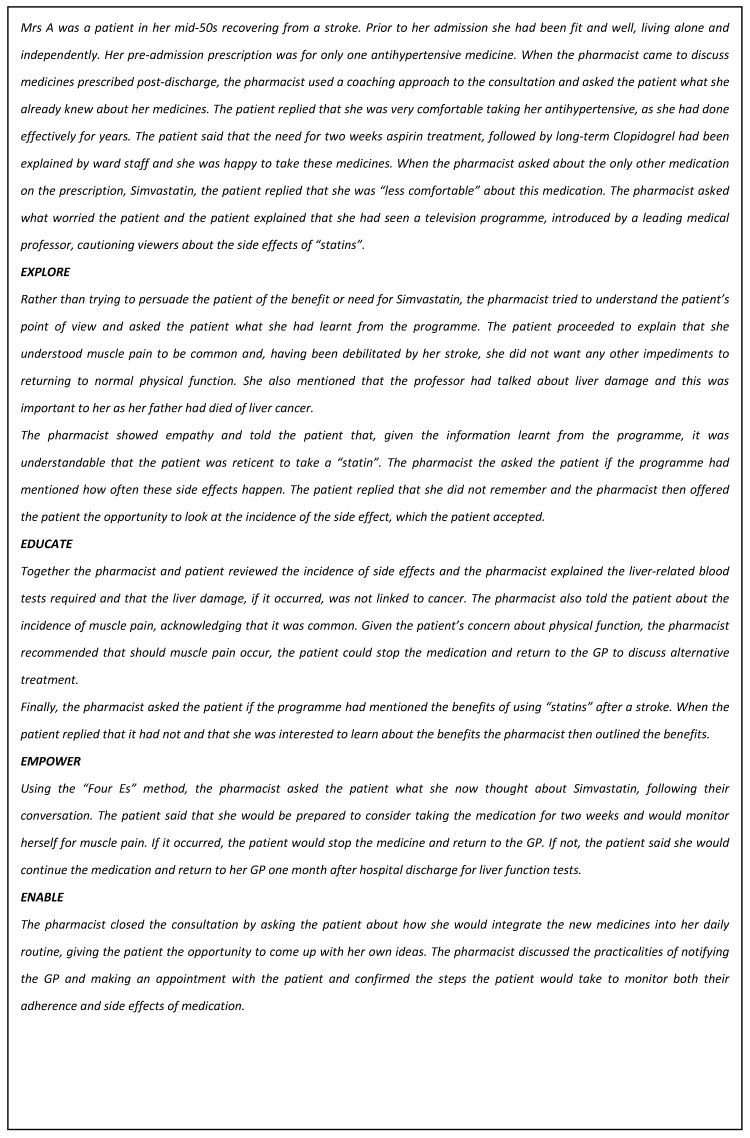
Illustrative example of the Four Es model in practice

A structure for short pharmacy consultations has been published [42], known as the “Four Es” (Explore, Educate, Empower, and Enable). The Four Es is a structure built on the “GROW” model [43], which is widely used in business and performance coaching and includes four elements (Goal, Reality, Options, and Will) to align with pharmacy structures for consultations [42]. The Four Es consultation structure [44] begins with identification of the topic of conversation which is usually introduced by the pharmacist, contrasting with a coaching conversation where it is identified by the “coachee”. Rather than the commonly-used method of the pharmacist telling the patient what they expect the patient to want or need to know and giving safety information, the Four Es require the pharmacist to use the “Explore” questions to identify the patient’s agenda. Education about medicines is delivered in line with the questions asked by the patient and safety information may be added where relevant. The patient is then asked, using a non-judgmental approach, to consider their decision in the context of taking the medicine, which is highlighted by the “Empower” stage of the structure. Once a decision has been made, the pharmacist supports the patient decision using the “Enable” questions, where the pharmacist helps the patient to think about what they need to do to put their decision into practice and how they will maintain and monitor their chosen path, to support whatever changes are agreed around health improvement. This may include lifestyle or diet changes, in addition to or as an alternative to medicines. The following example (see Figure 1) illustrates the use of the Four Es in practice. 

The use of this model to support medicines adherence was included in a review of current thinking about medicine adherence, which acknowledges the gap between addressing practical and perceptual approaches to support medicine adherence [45]. A coaching approach to pharmacy consultations, including the Four Es structure to consultations is now being used in local practice by pharmacy staff in areas such as general medicine, care of older people, and HIV. The structure has had a national impact on pharmacy consultations skills through inclusion in the Centre for Postgraduate Pharmacy Education document on consultation skills [44]. This is now being taught in undergraduate and post graduate university courses in the UK and Ireland.

The benefit of a coaching approach to consultations has been illustrated in video-recorded consultations for the Centre for Postgraduate Pharmacy Education [46], and in the dispensary setting. This included using the coaching principles to develop a consultation structure suitable for very short (2–5 min) pharmacy consultations [38] enabling use of a coaching approach to more patients. Videos also demonstrate how a coaching approach can be integrated into pharmacy practice in short consultations [47,48]. 

## 5. Conclusions

New thinking and a different approach to adherence support is beginning to emerge, both through academic literature and also practical day-to-day support in the clinical environment. Psychological theory such as the COM-B model and TDF have revolutionized our thinking in terms of providing evidence-based adherence support specific to the root cause of an individual’s non-adherence. True progress in this important field can only be made if we abandon aspirations for a one-size-fits-all approach to adherence intervention and understand non-adherence as a complex health behavior with a myriad of influencing factors. Novel tools to support practitioners to identify an individual’s barriers to adherence are emerging [18,21], representing an exciting opportunity to integrate theory and practice. However, work is still required to establish which behavioral change techniques are most appropriate for delivery in routine care, and also how routine healthcare providers, such as pharmacists, can be encouraged and supported to adopt these approaches. There is much to be learned from exemplars of innovative practice, such as those using the health coaching approach as described in this commentary. Our challenge, therefore, is to integrate theory with practice and highlight the merits of moving on from traditional models of adherence support; exciting opportunities are ahead if we have the courage and confidence to think differently.
